# A Qualitative Study of 11 World-Class Team-Sport Athletes’ Experiences Answering Subjective Questionnaires: A Key Ingredient for ‘Visible’ Health and Performance Monitoring?

**DOI:** 10.1007/s40279-023-01814-3

**Published:** 2023-02-10

**Authors:** Alan McCall, Adrian Wolfberg, Andreas Ivarsson, Gregory Dupont, Amelie Larocque, Johann Bilsborough

**Affiliations:** 1 Arsenal Performance and Research Team, Arsenal Football Club, London, UK; 2 School of Sport and Exercise Sciences, Edinburgh Napier University, Edinburgh, UK; 3 School of Sport, Exercise and Rehabilitation, University of Technology Sydney, Sydney, NSW Australia; 4 Medical Department, Football Australia, Sydney, NSW Australia; 5 Weatherhead School of Management, Case Western Reserve University, Cleveland, OH USA; 6 School of Health and Welfare, Halmstad University, Halmstad, Sweden; 7 Department of Sport Science and Physical Education, University of Agder, Agder, Norway; 8 School of Sport and Exercise, Liverpool John Moores University, Liverpool, UK; 9 University of Ottawa Law School, Ottawa, ON Canada; 10 Geneva Graduate Institute of International Studies and Academy of International Humanitarian Law and Human Rights, Geneva, Switzerland; 11 New England Patriots, National Football League, Foxborough, MA USA

## Abstract

**Background:**

Athlete monitoring trends appear to be favouring objective over subjective measures. One reason of potentially several is that subjective monitoring affords athletes to give dishonest responses. Indeed, athletes have never been systematically researched to understand why they are honest or not.

**Objective:**

Because we do not know what motivates professional athletes to be honest or not when responding to subjective monitoring, our objective is to explore the motives for why the athlete may or may not respond honestly.

**Methods:**

A qualitative and phenomenological approach was used, interviewing 11 world-class team-sport athletes (five women, six men) about their experiences when asked to respond to subjective monitoring questionnaires. Interview transcripts were read in full and significant quotations/statements extracted. Meanings were formulated for each interviewees’ story and assigned codes. Codes were reflected upon and labelled as categories, with similar categories grouped into an overall theme. Themes were examined, articulated, re-interpreted, re-formulated, and written as a thematic story, drawing on elements reported from different athletes creating a blended story, allowing readers a feel for what it is like to live the experience.

**Results:**

Overall, four key themes emerged: (i) pursuit of the ideal-self, (ii) individual barriers to athlete engagement, (iii) social facilitators to athlete engagement; and (iv) feeling compassion from performance staff.

**Conclusions:**

Our main insight is that athletes’ emotions play a major role in whether they respond honestly or not, with these emotions being driven at least in part by the performance staff asking the questions.

## Key Points

**Table:** 

Team-sport athletes are initially attracted to engaging in subjective monitoring with curiosity and openness that it might help them to become a better athlete and are willing to consider answering honestly; however, through their behaviours and actions, performance staff can invoke either positive or negative emotions in the players, which ultimately drives their level of honesty.
Despite common sense dictating that performance staff should cultivate a trusting and transparent relationship with athletes as well as facilitate an engaging environment, our study shows the power that performance staff actually have to either positively or negatively affect their athletes and the consequences for subjective monitoring.
To claim that subjective monitoring is a ‘waste of time’ because athletes are not honest without demonstrating this claim through empirically derived evidence has hindered the scientific investigation of “why” athletes are honest or not. Performance staff should reflect on their own behaviours and actions when delivering subjective monitoring.
The findings of this study bring into question the concept of ‘invisible’ monitoring as it is currently defined as this contradicts what athletes actually want from athlete health and performance monitoring, opening up new possibilities for ‘visible’ monitoring.

## Introduction

In 2023, athlete health and performance monitoring continues to be one of the hottest topics in sports science and medicine (referred to herein after as ‘performance’) research as well as one of the most commonly used strategies by performance staff, for example, scientists, fitness coaches, doctors, physiotherapists, psychologists, etc. Athlete monitoring can include either or both ‘*objective’* and *‘subjective’* measures. Objective monitoring typically involves the use of technology and wearables to measure various surrogates of, for example, athletic performance (such as sports-specific fitness assessments), physiological levels (including maximal oxygen uptake, muscle force and power, heart-rate, etc.,) and biochemical status (by extracting blood, saliva, urinary markers, etc.,) . In contradistinction, subjective monitoring provides insight into psychosocial and psychobiological factors internal to the athlete such as mental fatigue, effort, perceived stress symptoms, well-being, and motivation [1]. These factors are typically assessed using self-report tools like the Rating of Perceived Exertion (RPE), wellness items, perceptions of pain, psychological readiness, etc.

Importantly, objective and subjective monitoring are not interchangeable and give very different information [1]. Objective measures typically fragment observations to allow quantitative precision of metrics in isolation, whereas subjective measures reflect the blended input of multiple channels of information internal to the athlete [2]. Unfortunately, in athlete monitoring, and particularly in our experience in professional team-sports, performance staff and researchers seem to be relying more and more on objective monitoring. This trend is worrisome because subjective information has been shown to reflect acute and chronic training loads with superior sensitivity and consistency compared with objective ones [3]. Even a concept referred to as ‘invisible monitoring’ has been proposed, albeit with well meaning, to “lessen the burden on athletes” [4]. However, the operational definition of invisible monitoring has been stated as “gathering as much information about the athlete, their performance and their current training status, without them even knowing you’re doing it, in order to answer coach or performance driven questions” [4]. Invisible monitoring implies removing the athlete entirely from the monitoring process, but with this approach the consequence could be amassing unnecessary data that does not even reflect how the athlete actually feels, thus, increasing the likelihood of making ill-informed decisions about athletes’ full spectrum of health and performance capabilities.

## Literature Review

Literature on subjective monitoring of athletes mentions the likelihood of athletes’ not giving honest responses, but the current literature is limited in its empirical justification of such claims [1, 2]. In a study by Neupert et al. [5] in which nine female sprint water-sport athletes were interviewed, the majority of interviewees said that they responded honestly to training-monitoring questions. On the other hand, interviews including eight athletes from various high-level individual and team sports by Saw et al. [6] revealed that half of the athletes admitted withholding the truth on occasions through fear of punishment for not filling subjective questionnaires. To our knowledge there are no studies investigating athletes from the world’s major professional team-sports, for example, association football, rugby, basketball, American football, and baseball.

While not in a sports context, psychotherapy research may offer some additional valuable insights to further explore this topic and extend the work by Neupert et al. [5] and Saw et al. [6]. Instead of ‘honesty or dishonesty’, psychotherapy typically discusses this as ‘patient/client disclosure’ and its counterparts, i.e., concealment, secrets and lies [7]. In a psychotherapy review, Farber [7] explains that all patients at least occasionally conceal information or lie, with concealment being far more common than outright lying, and most of the time patients are actually quite open and honest. Patients concealing information or lying tends to be spontaneous ‘of the moment’ reactions, with other factors affecting responses that include: patients’ general comfort level in revealing stressful information; the nature of the patients’ character type; therapists’ responsiveness to disclosures, their experiences of previous disclosures (i.e., were they helpful in the past), and demographic factors such as ethnicity and culture (i.e., different cultures with different norms about what is appropriate to disclose). These insights appear to correspond with the earlier work in sports [5, 6], but require further investigation to advance knowledge in the sports domain.

In the area of sports performance, there are also anecdotal claims of athletes being dishonest in response to subjective monitoring questionnaires by performance staff and researchers at conferences and on social media. However, for claims of subjective monitoring ‘not being worth the effort because athletes tell lies’ there are also some anecdotal claims of the opposite experience, where staff and researchers’ perception and experiences are that the athletes they work with do provide open and honest responses. Hence, to our knowledge, the gap in the literature is that there are few, if any, systematic studies particularly in professional team-sports to shed light on why athletes do or do not tell the truth, nor what would drive them to be honest or dishonest. We have no advanced, a priori, theoretical or empirical knowledge as to what is going on in the context of the athletes’ minds regarding what is motivating them to answer honestly or not, and this should be investigated to better understand the phenomenon and to guide practical strategies.

Although sparsely investigated in sports performance literature, there are some studies [6, 8–10] lending support to the notion that athlete monitoring strategies should ideally be easy and quick where athletes are educated on what is being done and why as well as being adequately communicated to in follow-ups about the information they provide. However, a survey published in 2022 by Neupert and colleagues [11] found that feedback processes from monitoring strategies were largely felt to be ineffective, with 44% of respondents stating that athletes did not receive sufficient feedback, and in some cases the collected data were never even discussed with the athletes and/or coaches. This further supports the literature gap about athletes being honest or not, as we do not why, only that they may be or may not be.

Given the clear gap in the literature about professional team-sport athletes being honest or not or indeed what motivates their level of honesty in response to subjective monitoring, the current debate in sports performance lacks scientific investigation, and, consequently, provides little value to performance staff and their team management. While it has also been suggested that athletes’ responses might be dependent upon the wording of the questions [12], we take a step back from questionnaire phraseology and the logistics of a protocol, and delve into athletes’ experiences of answering subjective monitoring questionnaires. Therefore, the focus for us is not on the data collection format of the monitoring process through the questionnaire itself, but rather it is an exploratory study about the perspective and experiences of the athlete at the receiving end of the process. What is it about the experiences of the athletes that makes them want to engage honestly or alternatively to withdraw and disengage? Hence, our research question is, why do athletes respond honestly or not when being asked to respond to subjective monitoring questionnaires? This should bring us more in direct contact with the athletes when being asked these questions.

## Methods

To improve transparency, the Standards for Reporting Qualitative Research (SRQR) [13] (21 items) and the Consolidated Criteria for Reporting Qualitative Studies (COREQ) [14] (COREQ) (32-item checklist) were applied.

### Research Team and Reflexivity

The present authors comprise one female (AL) and five males (AM, JB, AW, GD, AI). AL (Msc) is an experienced delegate working and researching in the humanitarian sector, skilled in international law, foreign affairs and human rights. AM, JB and GD are PhD sport scientists and researchers, each with over 20 years’ experience as staff and researchers in world-class-level team sports. This experience includes leading and/or consulting in performance and research departments, and being part of national and international championship winning teams including men’s French, Scottish, Spanish leagues, National cups, UEFA Champions League, FIFA World Cup and NFL Superbowl. AI is a PhD psychology researcher and sport psychology consultant practising in world class and elite level sports teams. AM, JB, GD and AI are all experienced in conducting and publishing quantitative research, while AI is also experienced in qualitative research methods. AW is a PhD phenomenologist and qualitative researcher with almost 20 years’ experience working in the field and research in the organizational behaviour area.

Authors AM, JB, GD and AI have experience with subjective monitoring, which has been overall positive in regards to obtaining engagement from athletes. However, we also clarify that in our experience this is not automatically positive; we have had to work hard and consistently to get the buy-in from athletes, some of whom can be particularly argumentative, dismissive or unengaging. However, our experience ‘selling’ subjective measures in practice has taught us that obtaining honest responses from athletes relies not only on the athletes answering but also how we as staff approach subjective monitoring. What drove the initial idea to investigate the phenomenon of subjective monitoring were the differences in AM’s typically eventual, overall positive experience with subjective athlete monitoring, while being aware of the constant reports of negative experiences and criticisms from different groups in practice, research, social media and, anecdotally, at conferences, etc.

### Study Design

A phenomenological approach was chosen as the most appropriate using a series of one-to-one interviews to investigate the phenomenon of ‘athletes experiences when being asked to respond to subjective athlete monitoring questionnaires’, for example, s-RPE, wellness, sleep, fatigue, muscle soreness, psychological readiness, etc. *Phenomenology* is powerful in helping to understand a person’s experiences, which in this current study design involves the athletes’ experiences.

### Sample Selection

Purposive sampling was chosen to identify and invite persons to be interviewed. To determine who to include in our sample, we used our own professional network and knowledge of team-sport athletes and sports staff—both performance staff and technical/tactical coaches—who we knew were working with athletes, and who would be willing to participate as interviewees. Because the study’s context is the application of the sports performance role in professional sports, we targeted athletes competing in the major professional team sports, i.e., Association Football, American Football, Basketball, Major League Baseball, Rugby Union and Rugby League. To achieve some diversity in the sample, we sought to include interviewees with varying experience levels by inviting those in any of the following three career stages: (1) world class youth level, i.e., competing at international team level; (2) world-class in the ‘prime’ of their careers, i.e., currently competing as first-team regulars in the best league and international competitions in the world, winning or at least being finalists in at least one major tournament defined as a “one time sporting event of an international scale organized by a ‘special authority’ and yielding extremely high levels of media coverage” [15]; and, (3) world-class but recently retired, i.e., having competed as first-team regulars in the best leagues and international competitions in the world, winning or at least being finalists in at least one major tournament and being chosen as the ‘best player’ in their sport at national or international level at least once. We therefore excluded athletes who did not have extensive experience in the phenomenon under study—responding to data collection efforts to assess subjective measures. We followed the criteria and decision-tree to qualify as a ‘world-class athlete’ defined by McKay and colleagues [16]. After identifying potential athletes, we either contacted them directly or via colleagues in our network. We aimed to continue athlete interviews until we deemed saturation was reached, i.e., when interviewees introduced no new perspectives on the topic [17].

Eighteen world-class team-sport athletes were invited for interview. Invited athletes included seven athletes from women’s sport teams and 11 from men’s sports teams, competing in the top leagues of countries covering six continents. Represented team-sports included association football (four female, four male), American football (one male), basketball (one female, one male), baseball (one male), rugby union (two female, two male), rugby league (two male). Four (one female, three male) invited athletes were recently retired (within 15 years), and two were youth athletes (one female, one male, both over 16 years old but less than 18 years old).

Altogether, 12 athletes accepted to be interviewed; however, one was excluded as the athlete’s level in English speaking made it difficult to conduct the interview in the same way as with the others. Therefore, in total, 11 athletes were interviewed and the transcriptions of their interviews included for thematic analysis. See Table 1 for athlete demographic profiles.

**Table 1:** Overview of athletes’ profiles; demographics and athletic honours

Sports (men’s & women’s)	Nationalities (by continent)	Playing status	Age, y	Honours (major honours shown only)
Association Football, American Football, Basketball, Rugby Union, Rugby League	Europe, North America, South America, Asia, Oceania, Africa	Current, youth, retired	Mean: 31.1 ± 7.3	FIFA World Cup Champion, English Premier League Winner, UEFA Champions League Winner, UEFA Europa League Winner, Copa Libertadores Campeon, NFL Super Bowl Winners, NBA World Championship Winner, Africa Cup of Nations Winner, Rugby League World Cup Winner, National Rugby League Champion, Rugby League State of Origin Winner, Rugby League Club World Cup Winner, Rugby Union World Cup Runner Up, Olympic Gold, Silver and Bronze Medallists

### Setting

Athletes were interviewed online using Microsoft Teams video call, and the appropriate day/time was negotiated to coincide with their schedule. The actual setting of athletes varied from in their own home to a training facility or team hotel prior the morning of a competitive match. Interviews were not audio or video recorded. The intention to record an interview can influence the decisions interviewees take about the information they share [18], and an effective interview is in part about enabling an environment in which interviewees feel comfortable to say what they want [19]. Indeed data quality with appropriately trained interviewers between audio-recorded transcripts and interview scripts written directly after an interview have been shown to be comparable in the detail captured [18]. Given the high-profile nature of the athletes—i.e., world-class with significant media attention surrounding them, and that, with the exception of one of them, they did not have any prior knowledge of or relationship with the interviewer(s)—it was decided by AM, JB and AI that athletes would likely be more open and willing to be ‘interviewed’ without a recording. Hand-written notes were taken during the interviews and typed up in their entirety within 30 min of completing the interviews. AM conducted nine out of the 11 interviews and two were performed by two alternative interviewers, not in the principal research group. While one athlete was proficient in English, this person requested to be interviewed in their mother tongue to ensure they fully understood and in return were understood by the interviewer and optimizing a two-way conversation. In the other instance, the athlete did not speak English. For these interviews, the additional interviewers performed a pilot interview with AM to ensure interviews and questions were structured, delivered and performed in as close a manner as AM would have done. These additional interviewers were fluent in English and translated from the native language to English after the cessation of the interview with the athletes. These two additional interviewers comprised a sport scientist and a sports physician experienced in working with world-class athletes and in scientific research. As with the principal research group, both had similar experiences and assumptions regarding subjective monitoring. Neither had any prior relationship with the athlete they interviewed. Interviews lasted between 30 and 45 min.

### Data Collection Methods and Data Collection Instruments and Technologies

The interviews took place over a 1-year period from March 2021 to March 2022. An initial semi-structured interview guide was prepared taking into consideration aspects important for interview design [12]. The initial semi-structured interview was prepared by AM and AI and piloted with two athletes who were not involved in the study and known to AM. No changes were made to the initial interview guide.

Typed electronic records of the interviews were transferred onto Microsoft Excel. Files comprised separate columns where important full texts of interviews were winnowed to extract ‘quotations/statements’ deemed to be important and of interest, with additional columns prepared for the thematic analysis. Data analysis included (i) *first pass*: creating a code, (ii) *second pass*: converting the code to a category, and (iii) *determination of each category into an overall theme*, which is explained below, in data analysis. All raw interviews and participant information were de-identified and stored securely on Microsoft OneDrive by AM. Both AM and AI had access to a secure, private OneDrive shared folder.

### Data Analysis

Deriving findings from the interviews requires recovering a theme(s) that is embodied and dramatized in the evolving meanings of the work [20]. The specific process performed by AM was based on accepted guidelines for qualitative research analysis [20, 21]; *First*, the interviews were read in full to acquire a feeling for their ideas and to gain a deeper understanding. *Second*, significant quotations/statements were extracted by identifying key words and sentences relating to the phenomenon being investigated. *Third*, meanings for these statements were formulated. This process was repeated for each of the persons’ ‘stories’. *Fourth*, the quotations were re-read and reflected upon for each person separately, and a code assigned by writing a short sentence. *Fifth*, these short sentence codes were reflected upon to assign the most appropriate descriptive wording and labelled as a ‘category’. *Sixth*, a small number, typically five to eight, of overall ‘themes’ were generated to be shaped into a general description of the phenomenon, i.e., essence description [22, 23], displaying the perspectives of the persons.

After this six-step process was completed, the quotations, codes, categories and themes generated were reviewed by AI, who created notes where clarification and further discussion were needed. AM and AI then collaboratively reflected on and discussed each theme, category, code and quotation. Themes and their relevant categories were then further reviewed by AW, who collaboratively reflected with AM and AI to generate the final agreed upon themes. The themes were then examined, articulated, re-interpreted and re-formulated [22], and eventually written as a thematic story, drawing on elements reported from different athletes to create a blended story, allowing the reader to get a feel for what it is like to live the experience [24].

Steps have been taken by our research team to maximize and demonstrate the validity of the study [20]: (i) *Member checking* where a final report was returned to interviewees to determine whether or not they felt it accurately reflected their experiences and the insights they gave. (ii) *Clarifying the bias of the researcher(s)—*in the ‘reflexivity’ section of our article we clarify the potential *bias* that principal researcher AM and the research team may bring to the study through their own experiences and beliefs. (iii) *By presenting negative/discrepant information*, i.e., in the narrative we discuss ‘surprising’ codes that went contrary to our potential bias. (iv) *Spending prolonged time in the field—*we have provided earlier, a detailed overview of our credentials working in the practical setting, which demonstrate our in-depth understanding of the phenomenon under study. (v) *Peer debriefing—*where an independent person is located to review and ask questions about the qualitative study so that the account resonates with people other than the researcher. (vi) *An external auditor—*as distinct from a peer debriefer, the auditor is not familiar with the researcher or the project and provides an independent ‘peer’ review of the project.

## Findings

**Fig. 1:**
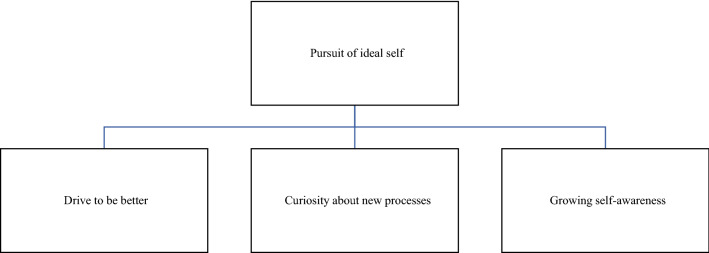
Factors supporting the theme ‘pursuit of ideal self’

**Fig. 2:**
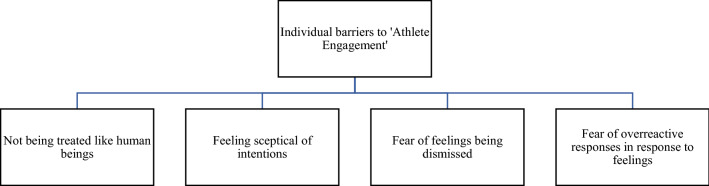
Factors supporting the theme ‘individual barriers to athlete engagement’

**Fig. 3:**
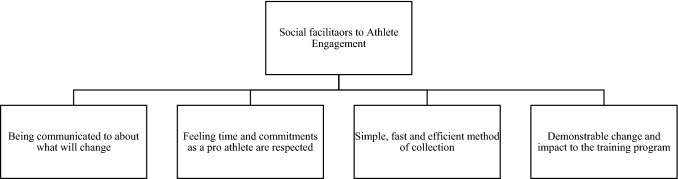
Factors supporting the theme ‘social facilitators to athlete engagement’

**Fig. 4:**
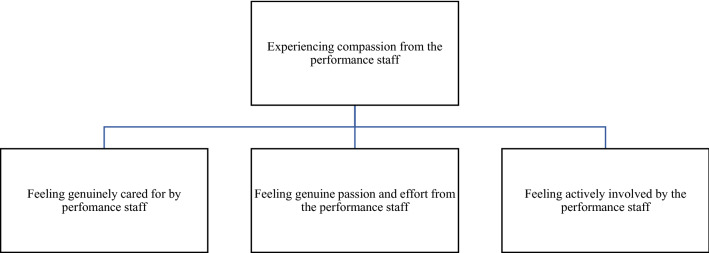
Factors supporting the theme ‘experiencing compassion from the performance staff’

Four themes emerged from the data analysis of the interviews. These were pursuit of ideal self, individual barriers to athlete engagement, social facilitators to athlete engagement, and experiencing compassion from the performance staff (see Figs. 1, 2, 3 and 4). The ensuing text describes each of these themes with quotations from athletes used to support the athletes’ claims, illustrate ideas, and illuminate experience [25].

### Pursuit of Ideal Self

‘Ideal self’ is a driver for intentional change [26] and was a key theme of discussion by athletes, i.e., the athletes’ preferred future regarding their professional sporting goals. Their pursuit—their motivational drive to improve and become a better athlete and curiosity about what they can do to achieve this—was described by all of the interviewees as an important part of their reasoning when deciding how to respond to subjective monitoring questionnaires. The pursuit of the ideal-self theme is grouped into three categories: drive to be better; curiosity about new processes; and growing self-awareness. Quotes from three of the athletes expressing this motivational drive are:

- “*We are so competitive, if we think something will give us an edge and we don’t take it, then there is something seriously wrong”* (A7).
- *“I am a player who always took care of my body and I understood quickly that this type of information was for my benefit” (A5).*
- *“I'm an inquisitive person, I like to know why we are doing things. The more I know for myself, I can put it into my own regimen and learn how to care of myself”* (A4).

Athletes also described the importance of recognizing that their real, current self evolves over a career as they become more aware of themselves, their bodies, and their goals. Several athletes discussed how, in general, their approach has been one of being willing to answer honestly. They understood that some athletes may be reluctant to give honest responses depending on each individual persons’ circumstances and past experiences, and acknowledged that they themselves had experienced similar reluctant feelings throughout their careers. One athlete said:

- *”How players respond depends on their experience and age, like the younger and older players…not everyone recognises that being tired or finding a session hard is just a normal part of training and being an athlete.. you learn this with experience” (A8).*

Another athlete had a similar reflection:

- *“I can see how some people could give a dishonest answer but it comes down to that person and being professional” (A1)*.

Two athletes described the change in response pattern as:

- *“How you respond to changes throughout your career, depending what stage you are at the more honest you will be” (A10).*
- *“I’m old enough now to know that they’re [staff] not going to make me run more [i.e., based on the response they give], as I get older I get to understand the process better, once we get to that point it’s easy to be honest” (A11).*

### Individual Barriers to Athlete Engagement

Individual barriers to athlete engagement can refer to intrapersonal aspects related to negative emotions, such as the negative effect of fear, and perceptions of doubt and scepticism about handing over personal subjective information about how they are feeling. More specifically, fear about mistreatment or dismissing of the athletes’ responses, i.e., their own data, is among the strongest mechanisms for the unwillingness to engage with honest responses to subjective monitoring questionnaires. The individual barriers theme is grouped into four categories: not being treated like human beings; feeling sceptical of intentions; fear of feelings being dismissed; and fear of overactive responses in response to feelings.

Athletes consistently described the importance of feeling like they are being considered and treated as human beings, where their own unique concerns, fears, desires and needs are considered, and the awareness that they are dealing with their own ‘things’ inside and outside of the sporting team. As an example, two athletes explained the importance of not being recognized as individual human beings:

- *“Acknowledge who people are, recognise and celebrate people, their cultures, families, what’s important to them. We have a lot of nationalities, Pacific Islanders, English, Irish, Australians…, if you can make them feel important and that the organisation is a family, they will get a sense of belonging and buy in to what the organisation is trying to do”* (A7).
- *“Understand what else is going on and respond to the circumstances like travel and other stresses [family, social]….you can't tell different types of stress with the external load sensors, what about family, social stresses, travel, too”* (A4).

The concept of ‘invisible monitoring’ came up in the interviews. Upon learning what this strategy entails, one athlete reacted with some confusion that this was actually used in teams:

- *“I’d feel like a robot. You need to know people care for you. Don’t try and take the human out of it…. Human interaction is so important”* (A2).

The second athlete discussing this concept reacted angrily:

- “I’m not a f**king science experiment, invisible monitoring to me, is cr*p, it’s my body, I need to know what is going on… these staff only care about themselves, making themselves look better and getting an increase in salary”. (A11).

Although treating athletes as the individual human beings that they are was described as being key to getting honest responses, it was acknowledged as a potential limitation in certain circumstances:

- “*The human aspect* [to subjective monitoring] *is definitely a limitation…. If we are angry, you’re more likely to get a short or very reactive reply… but human interaction is so important, especially when it’s someone I like to talk to"* (A2).

There was a feeling of being sceptical about the intended use of the information, for example, being used against them before their contract negotiations, or match selection, when asked through subjective monitoring. This scepticism played prominently in athletes’ minds when deciding whether or not to respond honestly. Scepticism was particularly heightened when questionnaires were introduced without prior warning or consultation with the athletes themselves, for example, through formal or informal communications, education sessions, etc.

One athlete reflected,

- *“I ask myself how honest should I be?…how is this going to be used?”* (A4).

Another athlete described their thoughts when being asked;

- *“I’m always trying to be honest, but I’m also thinking how trustable are the staff?”* (A9).

A third athlete stated:

- ”If it’s something you’ve never asked me before or I don’t know you, I’ll be like ‘what the h*ll’… is ‘xxxx’[e.g. the coach] going to see this?… if you rock up randomly and ask me these questions, I’m going to think ‘this is a bit weird’, what are you going to do with my information? (A8).

Another reason for the scepticism, especially without information about how the results are used, was related to the perceived impact on the chance of playing. One athlete stated that:

- *"We might think that we won’t train or play depending on how we answer”* (A5).

Another athlete reflected:

- “*Some players might not be comfortable with them [the staff] because they are wary of them” (A10).*

One athlete discussed a specific experience they had where their subjective information had been used against them:

- *“I’m pretty good at keeping my cards close to my chest, in x [the league], people [performance staff] can be so variable across the league and other teams, not many are in it for us [i.e. to improve the players’ health and/or performance levels], those guys in [the team], they’re there to sell themselves first and then worry about me, they want a better contract so they used the information to make themselves look better… they definitely presented everything negatively about me at the end of the season, they used it to make them look better; ‘he’s lazy, he’s out of shape, he hasn’t been reporting properly… I’ll now go to the GM [General Manager] and tell them if I don’t trust the staff and that he [i.e. the staff] might not trust me but just so he’s [the GM] aware there might be some mismatches in what he’s told” (A11).*

A fear of their feelings being dismissed or staff over-reacting also drove their thought process about whether or not to respond honestly. One athlete expressed:

- *“Your reaction to our responses is important, if we can tell that you are not taking us seriously we won’t answer properly… don’t antagonise us and don’t judge our answers”* (A2).

Another athlete described a specific experience of perceiving their feelings being dismissed at times when not in the starting squad for a match and being asked to rate how hard sessions were and how they were feeling:

- “If you’re in the practice squad, they [performance staff] wouldn’t give a s**t, they [performance staff] would only care about the guys who were going to be playing, so why even ask us because whatever we said it didn’t even matter” (A11).

### Social Facilitators to Athlete Engagement

Social facilitators to athlete engagement was one distinct theme that emerged. Social facilitators refer to perceptions by the athletes based on their experiences and beliefs about what staff within the performance team do, and/or can do to improve athlete engagement through facilitating the provision of information/data. These social facilitators are grouped into four categories that reflect different types of preferred actions: being communicated to about what will change; feeling time and commitments as a pro athlete are respected; simple, fast and efficient methods of collection; and demonstrable change and impact to the training program.

Being informed prior to the implementation of subjective questionnaires and educated about what change(s) will happen based on their information was highlighted by athletes as a key element of obtaining honest and accurate responses. One athlete, for example, discussed this importance especially when players are not familiar with subjective questionnaires:

- *“Professional players are not used to it [answering scales], if they didn’t start these when they were youth players… How players respond probably depends on their age and experience, like the young and older players… not everyone recognises that being tired or finding a session hard is just a normal part of training and being an athlete (A5).*

About the relevance of educating the athletes another reflected:

- *“Educating us on what you are doing and why would help us understand and more likely then to buy into it…expose the next generation to the methods, and the work you are doing” (A8)*.

Similar reflections were provided by another two athletes who stated:

- *“We want to know why we are doing things, to see how we are feeling and if something needs to be changed (A4).*
- *“Educate the players why you’re doing it, xxxx [the head of performance) did a lot of talking to everyone, as a group, to the individual players… It’s a lot of trial and error, trying to get the guys to do it… be persistent but you have to learn the persistence needed for each player… explaining on an individual level is important, explain to us the context, what it means to us [i.e. each individual player], how it will benefits us, you’re doing it to make our career better, little things like that” (A11).*

Athletes discussed their feelings and reactions to either experiencing no impact or experiencing positive impact, for example, changes to the training programme, based on the information they provide in subjective monitoring questionnaires. Having a demonstrable meaning or purpose to the information they are providing was key to giving honest responses. One athlete said:

*“The biggest thing is why? What changes are happening”? (A1)*.

When there are no meaningful changes or a positive impact to the programme, athletes described the following scenarios;

- “*Poor quality information or lack of practical information in a simple way we can understand are the main reasons why in my opinion, players do not answer correctly [i.e. with deliberately misleading responses]” (A5).*
- *”If you don’t come to see me, or I don’t see changes to my program or preparation I’ll just put anything. If you don’t react to the questionnaires, then I’m done…. I have experience where staff don’t follow the results… then I am not honest all of the time” (A3).*
- “I need to see validation of what they [staff] are doing with the results, if nothing changes from 2 weeks before I can tell you don’t give a cr*p…I’ll then just give you a different score than I really feel, probably like 1 or 2 points different just because I know you are expecting something to be different, but it’s not really how I feel [i.e., deliberately misleading response]” (A11).

However, when positive changes are seen and felt by the athletes, they were more prone to buy in to the process and give honest responses. One athlete stated:

- “*If my information is acted upon [i.e. used to improve the training program] then I’ll tell the truth, regardless of the person” (A3).*

Another athlete explained:

- *“…we want to see that our workouts are adjusted based on our feedback… as soon as we understood that it’s impacting our personal programmes, we were much happier to buy in…. as it gets more consistent we become more likely to be honest” (A2).*

A third athlete expressed:

- *“The staff need to use the information and communicate it back to us… like 15 min pre-meeting in the morning. It would be good to get feedback from the coach if they change something based on how we are feeling, like ‘you boys are tired so we changed this or that'” (A9). *

A strong desire for the subjective monitoring to be seamlessly integrated into the overall training programme was discussed by the athletes. In general, most athletes’ experiences centred around how quick, simple and timely the questionnaires are and that they believe the staff genuinely respect their time and other commitments they have as professional athletes. Three quotes from the athletes illustrating this:

- “*Overdoing it especially the wellness questionnaire can be a bad thing… if it’s too regular I would give the same answers or maybe only differ by one point”* (A9).

Another athlete explained,

- *“I don't want to be annoyed every morning by someone asking me all the time, like a nagging thing”* (A1).
- “*The simpler you make things for us, the better, we have enough going on being a professional player” (*A10).
- *“You maybe have about 5% of our time with our full attention, everyone’s trying to get to us, so make the most of it” (A11).*

Interestingly, it also shone through that an ‘integrated process’ is subject to individual athlete preferences;

- *“It definitely depends on the individual person*” [about how they will respond] (A1).

Although most athletes preferred to be asked subjective questionnaires in-person, this was not the case for everyone, as illustrated by three of the athletes below:

- *“I prefer the phone [to answer wellness questionnaires], as we are always on our phones, and it’s easy” (A1).*
- *“I prefer the app, it’s in my own time, it becomes routine, it’s just me and I can be totally honest” (A10).*
- *“I like when people talk to me, I feel like they care”* (A2).

### Experiencing Compassion from the Performance Staff.

Athletes expressed feelings about needing and experiencing compassion from the performance staff to be key to getting honest responses to subjective monitoring. The theme of compassion is grouped into three categories: feeling genuinely cared for by performance staff; feeling genuine passion and effort from the performance staff; and feeling actively involved by the performance staff, in particular, where the sports staff are able to demonstrate their passion, work ethic, commitment and authenticity to the individual athletes and the group as a whole. One athlete expressed the importance of relationships:

- “*Build relationships with us, we want to see how passionate you are about your role in the organisation…. then you will gain our respect” (A7).*

Another athlete highlighted the importance of empathy:

- *”Build relationships and convince us that you have our best interests at heart” (A10).*

The importance of empathy and recognizing the emotional state of the athletes at the time of being asked and how these questions may stir specific emotions was emphasized by two athletes:

- “*Emotions talk… if we are angry, you’re more likely to get a short or reactive reply” (A2). “One negative thing is that for wellness questionnaires it can bring the athletes mind to a particular soreness, all of a sudden I’d be drawn to focus on that hammy [hamstring] soreness and amplify it.. does it feel worse because I am focusing on it now?” (A7).*

A feeling of consistency was often described by athletes as a key part of their decision to be accurate and honest with regard to subjective questionnaires as well as their experience and growing self-awareness. This same consistency was highlighted as also being able to recognise potentially negative emotions and to answer without giving in to these.

- *“Emotions talk, as it gets more consistent [i.e. the whole subjective monitoring process] we become more likely to be honest” (A2).*

The susceptibility of responding to someone with whom you do not have a good relationship was exemplified by one of the athletes:

- *“I’ll be honest with my national team coach because I trust him, but I’ll ask if he is giving the data to [name] in my club team, because I don’t want him to see it, I don’t know what he does with my information” (A7).*

The ability of the staff to create an environment where the athletes feel at ease, genuinely cared for, and involved in a conversation about their subjective feelings helps athletes to believe that the persons asking the questions have their best interests at heart. This was stated by several of the athletes:

- *“It’s nice to know people care for you, don’t try and take the human out of it” (A2).*

Another athlete expressed:

- *“I appreciated that staff were listening and taking an interest in how I was feeling or any complaints I had….this made me feel comfortable to invest and answer honestly” (A5).*

One athlete explained that, as long as a trusting relationship had been built with the main person responsible for acting on the information, they would be honest, no matter who asked them the subjective questions:

- *“I would tell anyone [an honest response] because I knew they would tell xxxx [the head of performance] anyway and I knew he was in it for us and would use the information to make us better” (A11).*

Also the importance of involving the athletes in the process was stressed. One athlete said:

- *”Other factors are involved, like a big game coming up.. if my hammies are sore I’ll probably want to water it down a bit, but if you speak to me, involve me in the process like saying ‘ok, how can we modify this training session to keep you fresh for the game” (A7)*

How staff can work to establish a high-quality relationship was illustrated by one of the athletes:

- *”Build person to person relationships, have a conversation while you’re in the gym, like ‘how did you feel there mate?.. it’s like you’re having a coffee with them’” (A7).*

## Discussion

The purpose of this study was to understand *why* world-class professional team-sport athletes—both men and women—are honest or not when asked to respond to subjective monitoring questionnaires. In our findings, we identified four themes regarding why athletes are honest or not in responding to subjective monitoring instruments. All four are related to the athletes’ emotions. Just because performance staff must deal with emotions does not mean that subjective monitoring cannot be valid indicators of performance. These emotional needs provide insight into what performance staff can do to address these needs and achieve meaningful results from subjective monitoring.

What our study reveals is that not only are athletes driven by emotion, but, just as importantly, these can be the direct result of the relationship between the performance staff and the athlete, highlighting the importance of this interaction. Table 2 and the section below divide the emotional needs into temporal and spatial ones that athletes possess and how performance staff can respond in order to be responsive to these emotions.

**Table 2:** Attending to athletes’ temporal and spatial needs

Dimension	Dimension type	Athletes’ emotional needs	Suggested performance staff response
Temporal	Present	Fear that data will be misused	Recognize existence of fear
	Future	Pursuit of ideal self	Encourage pursuit of ideal self
Spatial	Information flow	Opaqueness of performance staff process	Use transparency and feedback
	Interpersonal interaction	Lack of compassion from performance staff	Develop a cooperative relationship

### Attending to Athletes’ Temporal Needs

Athletes have emotional needs that occur temporally, in the present and are targeted towards the future, which need to be fulfilled to facilitate honesty in responding to subjective monitoring instruments.

#### Performance Staff Encouragement in Athletes’ Achieving their Future Ideal Self

The athletes interviewed described their pursuit to become the best athlete they can, which aligns with the concept of the ‘ideal self’. The ideal self represents the preferred future and importance of a person’s dreams or aspirations in motivating change or the development driver of intentional change in one’s behaviour, emotions, perceptions and attitudes [27]. Creating a positive vision can facilitate perceptions of hope [27–29], which in turn stimulates the parasympathetic nervous system, resulting in increased openness, cognitive power, and flexibility [27]. When the ideal self is envisioned by the individual, it can guide actions and decisions in a direction that facilitates improved self-satisfaction through articulation and direction towards the emergence of a new state of being with self-actualization as a core quality [26]. The athletes interviewed consistently described their own growing self-awareness as important in their evolution of becoming the athlete they want to become, i.e. realizing who they actually are at that present moment in time. In accordance with the ideal self, acknowledging the current, i.e., real, self, and the discrepancy between this and the ideal self, is a powerful motivator for change [26]. Feeling and believing that performance staff are doing their best to genuinely help them achieve their ideal self appears to be a strong motivator for honest engagement in subjective monitoring practices.

#### Performance Staff Mitigation of Athletes’ Fear in the Present

The athletes interviewed stated their own internal barriers that negatively affect their ability to be open and honest when responding to subjective monitoring questionnaires [26]. These barriers included feelings of fear that the information they provide may be misused and/or their responses may be dismissed as trivial, made fun of or misinterpreted, for example, performance staff over-reacting, as well as scepticism about the overall intentions of performance staff. Such feelings led athletes to describe potential but significant trust issues with performance staff. A major issue for performance staff is trying to obtain honest responses from athletes who have perceptions of fear. Such fear can alter a person’s perception of the environment to be more threatening than it really is, resulting in defensive or hostile actions, in the person being more likely to withdraw or inhibit new thoughts and alternative ways to approach a situation [26]. The feelings described by the athletes in our study correspond to a fear that links closely with the psychotherapy literature, where the most common self-reported motives for lies and concealment of information are to avoid both shame and the therapist’s over-reaction or that the therapist will not understand a particular issue [7]. The person’s—for example in our study, the athlete’s—experience, either good or bad, will drive how the person reacts in future [7]. Our findings demonstrate that claims of the proper wording of questions in subjective monitoring questionnaires as the basis for inaccurate responses due to misinterpretation is not the entire explanation for why answers may be dishonest, but rather that they relate to an intentional dishonest response. Our findings extend those by Neurpert et al. [5] that emergence of emotions such as fear may result in deliberate dishonesty and strongly suggest that a consideration must include the emotions invoked in athletes through the performance staff’s communication behaviours and actions with them.

### Attending to Athletes’ Spatial Needs

Athletes also have emotional needs that, when fulfilled, support honesty in responding to subjective monitoring instruments that occur spatially in the information flow and in the interaction between performance staff and athletes.

#### Performance Staff Transparency and Information Flow Feedback with Athletes

The theme ‘social facilitators for athlete engagement’ described by our athletes centred around their experiences and subsequent emotions invoked about how subjective monitoring is implemented and facilitated in their team. Athletes explained how the performance staff’s methods and approaches to subjective monitoring can drive concealment, honesty or outright lying, i.e., how performance staff affect athlete ‘engagement’ in the process. Engagement can be described as the simultaneous employment and expression of a person’s preferred, i.e., ideal, self in task behaviours promoting connections to work and to others, and personal presence, whether physical, cognitive, or emotional [30]. ‘Disengagement’ refers to the uncoupling of selves from work roles where people withdraw and defend themselves physically, cognitively, and/or emotionally [30, 31]. The athletes’ experiences suggest that performance staff do not always facilitate—intentionally or unintentionally—an engaging process, and/or an environment that fosters honest and open responses. Our findings support previous research that a process facilitating subjective monitoring should be simple, efficient and, by design, engaging. However, our study provides additional insight into ‘why’ it should be this way, i.e., it is an opportunity to elicit positive emotions that actually motivate athletes’ to be honest. Additionally, we demonstrate a deeper appreciation of the impact that transparency can have in convincing athletes to be honest. While research has suggested the importance of educating and communicating with athletes on what is being done in regards to subjective monitoring questionnaires, we show ‘why’ this is actually important. Specifically, a two-step process must occur: first, athletes need to experience and therefore believe that there is meaning/purpose to what they are being asked to do; and second, once they are convinced of the meaning, this then needs to be demonstrated through consistent behaviour and action of the performance staff through feedback and impact to training. Meaningfulness specifically refers to the extent that people derive meaning from their work and feel that they are receiving a return on investment, where they feel worthwhile, useful, valuable and not taken for granted [31]. This is essentially what the athletes in our study are seeking, and by doing so, the return on investment for the efforts of the performance staff themselves will be honest engagement; in other words, everybody wins.

#### Performance Staff Development of an Interpersonal, Cooperative Relationship with Athletes

Athletes' desire that performance staff treat them with compassion, represents an important behaviour of the performance staff that can elicit positive emotions through feeling convinced about staff intentions and therefore opening up athletes’ honesty. Compassion can be described as consisting of three principal components: (1) empathizing with the other, (2) caring for the other, and (3) acting in response to the other’s feelings [32, 33]. Essentially, compassion can be viewed as noticing another’s need or desire, and by ‘coaching them with compassion’, we are focusing on invoking the ideal self to initiate and guide the change process [33]. Compassion’s function is the maintenance of cooperative relationships [34], and to be successful, the coach, i.e., performance practitioner in our example, must establish and cultivate a trusting relationship with the athlete so they discuss their hopes and dreams openly, and develop in them a sense of safety to explore new thinking and development [35]. For the athletes in this study, an essential part of feeling compassion from performance staff was being actively involved in both the subjective and the overall monitoring process. While getting buy-in from players is not always easy, the trend to remove the athlete from the process, for example, through ‘invisible monitoring’, does not appear to correspond with how athletes see the full benefits of a health and performance monitoring program. Based on our results, it is more likely that ‘visible’ monitoring where athletes are actively involved, for example, through coaching with compassion, will arouse positive emotions and healthy psychophysiological systems helping them become more open to new possibilities, grow and renew themselves, leading to favourable outcomes at the individual, dyad, group and organizational levels [33].

## Limitations

For the reasons stated in the Methods section, the authors of this study made the intentional decision not to record interviews. It is always a risk doing so because not all information will be captured by the interviewer. However, the benefits outweigh this risk by acquiring thick descriptions of the phenomenon of interest. Consequently, we have taken several steps to maximize and demonstrate the qualitative validity of our findings and our interpretive discussion. We implemented ‘member checking’ to ensure the athletes interviewed felt our account of their experiences was accurate. We clarified researcher bias based on our own personal views and experience in the reflexivity section earlier in our article. Additionally, we included both a peer debriefer and an external auditor to review and provide feedback on the manuscript prior to submission. We also acknowledge that we focused specifically on world-class professional team sport athletes and the experiences of amateur or semi-professional team sport athletes as well as individual sport athletes and/or athletes competing at elite, amateur or recreational levels may have different and/or unique experiences that relate specifically to them. We also realise that we have interviewed athletes only, and interviewing of performance staff to understand their experiences and the potential mutual role that both parties might play should be explored.

## Practical Application

This study does not prescribe generalized methodologies, sets of techniques, or rules for acting as seen in ‘typical’ practical applications; rather, through an analytic way of thinking, we provide performance staff with insights that can strengthen the relationship between thoughtfulness and tact.

Cultivating trusting relationships with athletes and creating an environment that facilitates openness and honesty appear to be what athletes are seeking from performance staff. As performance staff we clearly need to have self-awareness around how athletes might perceive and experience our behaviours and actions toward them and our power to invoke either negative or positive emotions in them. We can elicit positive emotions through helping athletes to become the best athlete they can, i.e., to be their ‘ideal self’, by behaving and acting genuinely and with consistency in a way that convinces athletes that we are genuinely there to help them and not just in it for ourselves.

We should reflect on our subjective monitoring protocols, being aware about which questionnaire(s) we use, how we implement them and when we ask questions. These are more than purely ‘logistical’ matters, but rather correspond to how an athlete will react emotionally and dictate their responses. Ultimately, we should ask ourselves, are we truly caring for the athlete? Are we really acting in response to how each individual person is feeling in the present and caring for their future? Are we coaching them with compassion? Overall, performance staff being aware of and tapping into athletes’ pursuit of their ideal selves and accompanying them on their journey to bridge the gap between their real and ideal self may represent a potentially powerful strategy for staff to get honest buy-in from athletes.

## Future Directions

Our insights open up new and exciting areas for scientific investigation, in particular towards a deeper understanding of athletes’ pursuit of their ideal selves and how we can most effectively help them to transition toward their preferred future including their dreams, hopes and desires. This represents an exciting area for future research into athlete engagement and the role of emotions in providing honest responses. The implications are not only to be found in subjective monitoring but could be extended to the entire athlete preparation domain, and how we engage and build relationships with athletes throughout the entire health and performance process.

## Conclusion

While other qualitative methodological studies cited in this article have studied athlete perceptions, our study is one of the few, to our knowledge, to describe and attempt to understand the “why” of whether athletes respond honestly to subjective monitoring questionnaires. Our findings revealed that the honesty of athlete responses may be largely driven by the emotions invoked within them in response to the behaviours and actions of performance staff asking the questions, with negative emotions fostering dishonesty and positive ones encouraging honesty. Positive emotions are experienced by athletes when they are convinced that performance staff are genuinely doing their best to help them to become the best athlete that they can be, that their time and effort is being respected, and that there is demonstrable meaning to them participating in subjective monitoring processes.
